# Differentially Expressed tRNA-Derived Small RNAs Co-Sediment Primarily with Non-Polysomal Fractions in *Drosophila*

**DOI:** 10.3390/genes8110333

**Published:** 2017-11-20

**Authors:** Çağdaş Göktaş, Hatice Yiğit, Mehmet İlyas Coşacak, Bünyamin Akgül

**Affiliations:** 1 Department of Molecular Biology and Genetics, İzmir Institute of Technology, Gülbahçeköyü, 35430 Urla İzmir, Turkey; cagdasgoktas@gmail.com (Ç.G.); hatice.yigit@gmail.com (H.Y.); m.i.cosacak@gmail.com (M.İ.C.); 2 Unit of Molecular Cell Biology, Johannes Gutenberg-University Mainz, Bentzelweg 3, 55128 Mainz, Germany; 3 German Center for Neurodegenerative Diseases Dresden, DFG-Center for Regenerative Therapies Dresden, Cluster of Excellence, TU Dresden FetscherstraBe 105, 01307 Dresden, Germany

**Keywords:** tRF, tRNA, polysome, *Drosophila*

## Abstract

Recent studies point to the existence of poorly characterized small regulatory RNAs generated from mRNAs, rRNAs and tRNAs. To explore the subcellular location of tRNA-derived small RNAs, 0–1 and 7–8 h *Drosophila* embryos were fractionated on sucrose density gradients. Analysis of 12,553,921 deep-sequencing reads from unfractionated and fractionated *Drosophila* embryos has revealed that tRFs, which are detected mainly from the 5’ends of tRNAs, co-sediment with the non-polysomal fractions. Interestingly, the expression levels of a subset of tRFs change temporally following the maternal-to-zygotic transition in embryos. We detected non-polysomal association of tRFs in S2 cells as well. Differential tRF expression pattern points to developmental significance at the organismal level. These results suggest that tRFs are associated primarily with the non-polysomal complexes in *Drosophila* embryos and S2 cells.

## 1. Introduction

Transfer RNAs (tRNAs) are well-known for their role in protein synthesis [1]. tRNAs are also involved in priming reverse transcription in viruses, inducing gene expression under amino acid starvation and regulating splicing and apoptosis [2,3,4]. The intracellular dynamics of tRNAs is quite diverse among species [5]. tRNAs are subject to quality control mechanisms, such as nuclear RNA surveillance and rapid tRNA decay during biogenesis and maturation [6,7].

Broadly, two main groups of tRNA-derived short RNAs exist in the literature; tRNA halves (tRNA-derived stress-induced small RNAs, tiRNAs or sitRNAs) and tRNA-derived fragments (tRFs) [8]. sitRNAs are generated in response to stress through endonucleolytic cleavage by Rny1 and angiogenin ribonucleases in yeast and mammalian cells, respectively [9,10]. There is no specificity as to which tRNAs are cleaved under stress conditions. Additionally, no change, under stress, in the amount of the full-length tRNA levels suggest that the cleavage is a general response, not a regulatory mechanism. tRFs were often cloned during the early discovery of miRNAs through conventional cloning methods. The advances in sequencing technology have led to the identification of a plethora of tRFs [11,12,13,14,15,16]. More careful analysis of deep-sequencing data revealed a potential regulatory role for tRFs [12]. Conflicting evidence exists with respect to the biogenesis of tRFs. Earlier studies suggested that tRF production is Dicer-dependent [11,13]. However, recent work excluded the canonical miRNA machinery for the production of tRFs [17].

The studies to understand the role of tRFs in gene regulation mainly dealt with miRNA-like functions. It was proposed that tRFs may compete with pre-miRNAs for Dicer, modulating miRNA homeostatis [11]. tRFs were also proposed to modulate the silencing activities of microRNAs and siRNAs [14]. However, tRF-1001, which induces cell proliferation in a human prostate cancer cell line, apparently functions through a mechanism different from miRNA/siRNA pathways [12]. In *Tetrahymena*, tRNA fragments of 18–22 nucleotides are selectively bound by a specific Piwi protein, Twi12 [18]. In *Drosophila*, tRFs are associated with Argonaute 1 (AGO1) and AGO2 [16]. Both sitRNAs are tRFs are reported to inhibit cap-dependent translation initiation [19,20]. The conflict exists in the cytoplasmic location of tRFs as well. Studies conducted with a limited number of tRFs point to polysomal association of tRFs in human cells [20] but non-polysomal localization in *Trypanosoma cruzi* [21].

In this study, we fractionated 0–1 and 7–8 h *Drosophila* embryos to investigate the subcellular localization of tRFs at the genomics level. Our data show that tRFs are detected mostly from the 5’ ends of a subset of tRNAs, suggesting not only temporal but selective expression under physiological conditions. Based on their length and cleavage site, tRFs appear to be different from sitRNA fragments. tRNA-specific differential biogenesis points to the important developmental function of tRFs in higher eukaryotes. More importantly, tRFs are primarily associated with mRNP and 60S fractions, cytoplasmically localizing away from the actively translating polyribosomes.

## 2. Materials and Methods

### 2.1. Embryo Collection, Cell Culture and Transfection

*Drosophila* P2 strain was a gift of Dr. C.-P. David Tu of Pennsylvania State University. Flies were grown at 22–25 °C on standart yeast-sucrose-agar medium. Embryos were collected on embryo collection plates containing grape juice (0–1 h embryos) according to standart protocols. The embryos were stored at −80 °C until use. S2 cells, which were generously provided by Dr. Ylva Engström of Stocholm University, were grown in Schneider’s *Drosophila* medium at 25 °C. Cells were transfected with 0.08 mM biotinylated tRFs using the calcium-phosphate transfection kit according to the manufacturer’s instructions (Invitrogen, Waltham, MA, USA).

### 2.2. Polysome Analysis and RNA Isolation

Total RNA was isolated from S2 cells and unfractionated embryos using TRIzol^®^ (Invitrogen) and miRVana miRNA isolation kit (Ambion, Waltham, MA, USA), respectively. Ribonucleoprotein complexes were fractionated according to a previously published procedure with minor modifications [22]. Briefly, embryos (0.2 g) or S2 cells (3 × 10^7^) were homogenized in 5 mL lysis buffer ((100 mM NaCl, 10 mM MgCl_2_, 30 mM Tris-HCl (pH 7), 1% Triton X-100, 1% NaDOC, 100 µg/mL cycloheximide (Applichem, Darmstadt, Germany) and 30 U/mL SUPERase.In^™^ RNase Inhibitor (Ambion, Waltham, MA, USA)) and incubated on ice for 8 min. The homogenates were centrifuged at 12,000× *g* at 4 °C for 8 min. Two-mL supernatant was loaded onto 5–70% (*w*/*v*) sucrose gradients (100 mM NaCl, 10 mM MgCl_2_, 30 mM Tris-HCl (pH 7), 200 U SUPERase. In^™^ RNase inhibitor (Ambion, Waltham, MA, USA)) and centrifuged at 27,000 rpm for 2 h 55 min at 4 °C in a Beckman SW28 rotor. Fractions were collected using an ISCO density gradient system. Fractions were then pooled into 4 sub-groups based on their A_254_ readings; mRNP, 60S, monosome and polysome. Total RNA was extracted from the fractions as previously described [22]. RNA quality was assessed by 2100 bioanalyzer using RNA 6000 Nano Kit based on manufacturer’s instructions (Agilent, Santa Clara, CA, USA).

### 2.3. Small RNA Cloning

Small RNAs were cloned using miRNA cloning kit according to manufacturer’s instructions (Abnova, Taiwan). Fifty µg total RNA (or 200 ng small RNA enriched by mirVANA miRNA isolation kit (Ambion, Waltham, MA, USA) was used as the RNA input. 1/100th of the resulting cDNA was used for PCR amplification. An aliquot of the PCR products was cloned into pGEM^®^-T Easy vector and subjected to Sanger sequencing at the BIOMER core facility (Izmir, Turkey).

### 2.4. Small RNA Deep-Sequencing and Data Analysis

Three replicates of RNAs from unfractionated and fractionated (mRNP, 60S, monosome and polysome) 0–1 and 7–8 h embryos were mixed in equal amounts (in micrograms) and sequenced using Illumina Genome Analyzer GAII and chrysalis 36-cycle v3.0 sequencing kit by Fasteris (Switzerland). miscRNA, ncRNA, transcripts, transposons, intron, intergenic regions and pseudogene sequences were obtained from flybase (www.flybase.com), hairpin sequences from mirbase (Release 17) (www.mirbase.org) and 18S, 28S (extracted from M21017.1 GenBank) and 2S (rRNA) variants from NCBI. Nexalign program (http://genome.gsc.riken.jp/osc/english/software) was used to align small RNAs to *Drosophila melanogaster* (dmel-r5.39) genome including ChrU and tRNAs with 5’-CCA-3’ appended but not ChrUextra. The sequences were aligned first for exact match, then using remaining sequences for one (mismatch, insertion or deletion), two and three mismatches as described previously [23]. The order of alignment was as follows: rRNA followed by hairpin, tRNA (CCA appended), miscRNA, ncRNA, transposon, transcript (5’UTR-CDS-3’UTR), intron, pseudogene and intergenic regions. The data were deposited to GEO under the accession number GSE35443.

### 2.5. Northern Blotting

A pair of primers (Alpha DNA) containing a T7 promoter and a sequence of interest were heated at 95 °C for 5 min and incubated at room temperature for 30 min. The resulting double-stranded DNA was used as a template to generate antisense riboprobes in the presence of 0.5 mM biotin-conjugated UTP using MAXIscript transcription kit according to the manufacturer’s instructions (Ambion, Waltham, MA, USA). Total RNAs were resolved on a 12% denaturing polyacrylamide gel with urea and transfered onto a PVDF membrane (Ambion, Waltham, MA, USA). The nucleic acids were cross-linked to the membrane at 80 °C for 25 min. Pre-hybridization was carried out at 37 °C for 1 h in hybridization buffer (7% SDS, 200 mM Na_2_HPO_4_, pH 7.4). Riboprobes were incubated with the membrane at 37 °C overnight. The filters were then washed three times at 37 °C for 10 min with 2× SSPE buffer (300 mM NaCl, 20 mM NaH_2_PO_4_, 20 mM EDTA) containing 0.01% SDS. Washing was repeated at room temperature with 1X PBS (137 mM NaCl, 2.7 mM KCl, 10 mM Na_2_HPO_4_, 2 mM KH_2_PO_4_) containing 0.05% SDS. The signals were visualized with the supersignal west femto chemiluminescent substrate (Thermo Scientific, Waltham, MA, USA) after hybridization with streptavidin-conjugated horseradish peroxidase according to the manufacturer’s instructions. Images were acquired and quantified with Versa Doc gel imaging system (Bio-Rad, Hercules, CA, USA).

## 3. Results

### 3.1. tRFs Are Detected Primarily from the 5’-end Sequences of tRNAs in Drosophila

Substantial changes occur in the expression of miRNAs and siRNAs in *Drosophila* following blastoderm cellularization, which takes place between 2.5 and 3 h post-fertilization [24]. We investigated the intracytoplasmic distribution of small RNAs by subjecting embryonic extracts to small RNA profiling by deep-sequencing. During our analyses, we noticed fragments being produced specifically from tRNAs. Small RNA transcriptome analysis already unravelled the existence of small fragments from tRNAs in cancer-derived cell lines [11,13,15]. Our findings were of interest because it provided a genome-wide account of tRF sub-cellular location under physiological states (e.g., development). We deep-sequenced small RNAs from unfractionated and fractionated 0–1 and 7–8 h *Drosophila* embryos. Following the removal of the adaptor sequences and quality control analyses, we carried out our analyses with 15–29-bp fragments that perfectly match to the *Drosophila* genome.

**Figure 1:**
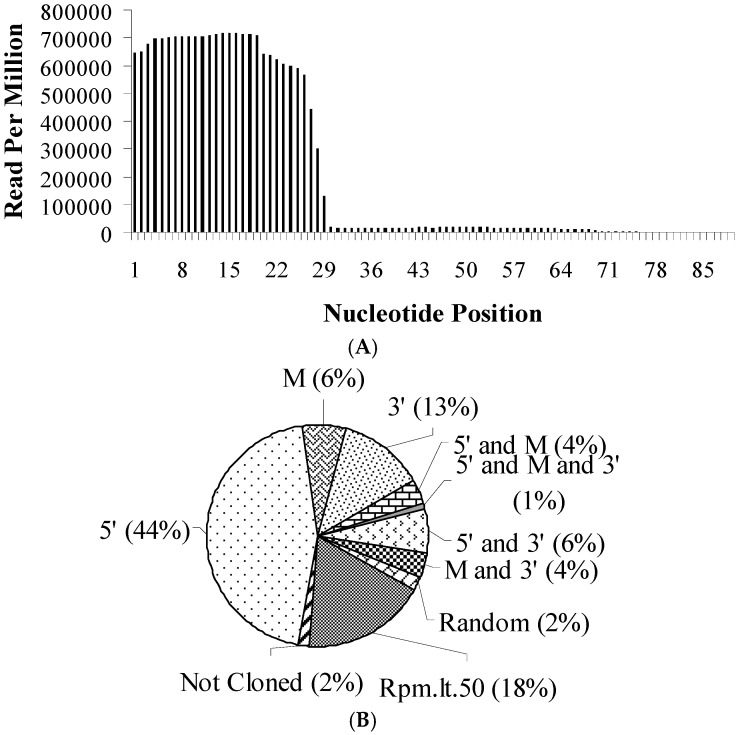
tRFs are detected primarily from the 5’-ends of tRNAs in *Drosophila*. (**A**) Cloning frequency of each nucleotide on a representative tRNA. All tRNA-derived fragments identified in unfractionated and fractionated samples were aligned to the tRNAs they originated from and the position of each nucleotide was determined relative to the 5’ end. The cloning frequencies of all nucleotides relative to the 5’-end were summed and graphed on a representative tRNA. (**B**) Percentage of tRNAs from which tRFs are generated. 103 tRNAs were folded into a cover leaf structure as determined by the tRNAScan-SE program [25]. Each tRNA was then divided into three main regions; 5’ (1–26 nt), M (middle, sequences flanking the region between the first and last 26 nt) and 3’(the last 26 nt). The frequency of each nucleotide position normalized to read-per-million was divided by the length of the fragments to obtain the relative expression ratio per nucleotide site on individual tRNAs. Based on the location of the reads, each tRF was assigned to a region on a reference tRNA (5’, middle (M) and 3’). For a tRF to be assigned to a tRNA region (e.g., 5’, middle or 3’), at least 2/3rd of the tRF must overlap that region. Rpm.lt.50—read-per-million is less than 50.

The sizes of the most abundant tRFs were 28 (25.1%), 27 (18.3%) and 26 (17.6%) nucleotides. When all tRNA-derived sequences were superimposed onto a representative tRNA relative to the 5’end, 95.74% of the reads aligned to the 5’ ends of tRNAs (nucleotides 1 and 28) (Figure 1A). These tRNA fragments are referred to as tRF-5 hereafter (e.g., tRF^gly:GCC:5^, tRF-5 derived from the tRNA carrying glycine amino acid with the GCC anticodon). It is noteworthy to state that, some tRFs aligned specifically to the mid sections or 3’ ends but with a very low cloning frequency compared to the tRF-5s. To quantitate the distribution of tRFs along the tRNA sequences, we divided tRNAs into 3 sections (5’, mid-section and 3’) and aligned tRNA-derived reads onto individual tRNAs. Of 111 *Drosophila* tRNA genes tested, tRF-5s were generated from the 5’-ends of 50 tRNAs (Figure 1B). Mid-section- and 3’-end-derived tRFs were produced from 7 and 14 tRNAs, respectively. No tRFs were detected from two tRNAs (tRNA:CR30260-RA and tRNA:CR30505-RA). The cloning frequency of tRFs from 20 other tRNAs was less than 50 RPM (read-per-million) while 2 other tRNAs were randomly fragmented. The remaining 16 tRNAs were processed to generate tRFs from a combination of 5’, mid- and 3’-end sections (Figure 1B). We selected the most abundant tRF species for characterization in subsequent analyses.

**Figure 2:**
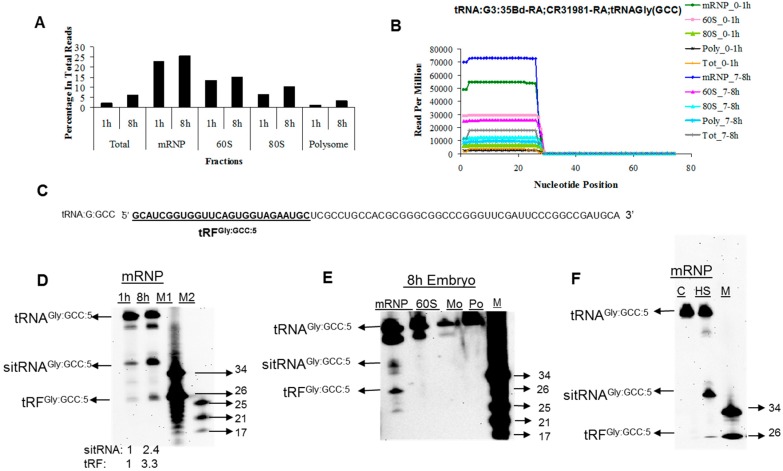
tRNA-derived fragments are associated with mRNP and 60S fractions. (**A**) Cloning frequency of tRNA-derived fragments in unfractionated (Total) and fractionated (mRNP, 60S, 80S and Polysome) samples. (**B**) Cloning frequency of each nucleotide derived from tRNA^Gly:GCC^. (**C**) Nucleotide sequence of tRNA^Gly:GCC^. The sequence of tRF^Gly:GCC:5^ is underlined. (**D**) Northern blot analysis of tRF^Gly:GCC:5^ in the mRNP fractions of 0–1 and 7–8 h embryos. Biotinylated antisense transcripts complementary to tRF^Gly:GCC:5^ in *C* was used for probing 5 µg RNAs from the mRNP faction. RNA loading was normalized against mature tRNAs and fold of induction of sitRNA and tRFs are indicated under each lane. M—Marker. (**E**) Northern blot analysis of tRF^Gly:GCC:5^ in 7–8 h embryos. 7–8 h embryos were fractionated on 5–70% sucrose density gradients (mRNP, 60S, mo (monosome) and po (polysome)) and total RNAs from each was probed with biotinylated antisense transcripts as in *D*. 4 µg mRNP, 6 µg 60S and 50 µg monosomal and polysomal RNAs were loaded to obtain approximately equal amounts of mature tRNA for normalization. (**F**) tRF-derived fragment (tRFGly^:GCC:5^) is expressed in S2 cells under physiological states. Fractionation and Northern blotting were carried out as in D–E. M—Marker; C—Control S2 cells; HS—Heat shocked S2 cells (4 h at 43 °C); sitRNA, stress-induced tRNA fragment. The sitRNA and tRF expression ratios are based on signal intensity.

Although the temporal and spatial expression of miRNAs during the maternal-to-zygotic transition (MZT) is well-documented [26,27,28], the expression patterns of tRFs during this process is unknown. Quantitative analyses of deep-sequencing data from unfractionated 0–1 and 7–8 h embryo total RNAs revealed several interesting points. Firstly, the overall percentage of tRF-5s in 7–8 h embryos is higher than that in 0–1 h embryos (Figure 2A, Total). Certain tRFs appear to be over-expressed in 0–1 h embryos (e.g., tRF^Pro:AGG:5^ 3.7 fold) while some others are down-regulated (e.g., tRF^Gly:GCC:5^ and tRF^mt:tRNA:S:AGY:5^ 4.6- and 18.9 fold, respectively) (Supporting Table S1). Secondly, tRFs are detected from a selective subset of tRNAs. For example tRFs are generated from tRNA^Gly:GCC^ and mt:tRNA^S:AGY^ at much elevated levels. Thirdly, we noticed selectivity among the tRNAs carrying the same aminoacid but possessing a different anticodon (Supporting Table S1, compare mt:tRNA^S:AGY^ and mt:tRNA^S:UCN^ in 0 to 1 and 7 to 8 h Total).

### 3.2. tRFs Are Associated with Non-Polysomal Complexes in Drosophila Embryos and S2 Cells

The temporal expression pattern and selectivity in detection of tRFs from the 5’ends of a subset of tRNAs suggest a biological function in early development. Since sitRNA fragments were proposed to regulate their targets translationally similar to miRNAs [9], we wanted to check whether tRFs interact with polysomes. We hypothesized that if tRFs are involved in translational regulation, they should be differentially associated with polysomes at pre- and post-MZT stages. Thus, we fractionated 0–1 and 7–8 h embryonic extracts into 4 major fractions based on their rRNA contents: (1) translationally inactive messenger ribonucleoprotein (mRNP, which also includes free RNAs), which is devoid of 18 and 28S rRNAs; (2) 28S rRNA-containing 60S; (3) monosome and (4) actively translating polysomes (Supporting Figure S1A–B). The QC analysis by capillary electrophoresis showed that the quality of monosomal and polysomal total RNAs was just as good as that of the total RNAs isolated from unfractionated embryos (Supporting Figure S1C). The sum of all tRF-5s was much higher in the mRNP fraction compared to the monosomal and polysomal fractions (Figure 2A). We then calculated the cloning frequency of individual fragments. This analysis has revealed that tRFs are primarily associated with the non-polysomal mRNP fraction (Figure 2B, Supporting Table S1 for other tRNAs). Except for a few monosome-associated tRFs, the cloning frequency is extremely low in monosomal and polysomal fractions. The 0–1/7–8 h ratio of cloning frequency is highly similar in unfractionated and fractionated embryos, further supporting biological functions associated with individual tRFs (Supporting Table S1).

We used conventional cloning methods to validate the presence of tRFs in the mRNP fraction of 2 h embryos. The RT-PCR amplification of adaptor-linked 35–40-nt small RNAs revealed that these RNAs are present in the mRNP fraction of 2 h-embryos as well (e.g., CR31540). Northern blotting analysis showed that tRF^Gly:GCC:5^, which is produced mainly from the 5’-end of glycine tRNA (Figure 2C), is highly expressed in the mRNP fraction (Figure 2D,E). We detected two major fragments in the sizes of 26- and 40 nt (Figure 2D,E). In accordance with the deep-sequencing data, tRF^Gly:GCC:5^ expression was up-regulated in the mRNP fraction of 7–8 h embryos although the expression level of the corresponding mature tRNA remained unchanged (Figure 2D). Thus, we normalized RNA loading based on the mature tRNA levels in the subsequent experiments. We observed similar results in S2 cells, a cell line derived from dissociated embryos at near hatching stage [29]. tRF^Gly:GCC:5^ was associated with non-polysomal fractions under stress (heat shock at 42 °C for 4 h) conditions (Figure 2F).

**Figure 3:**
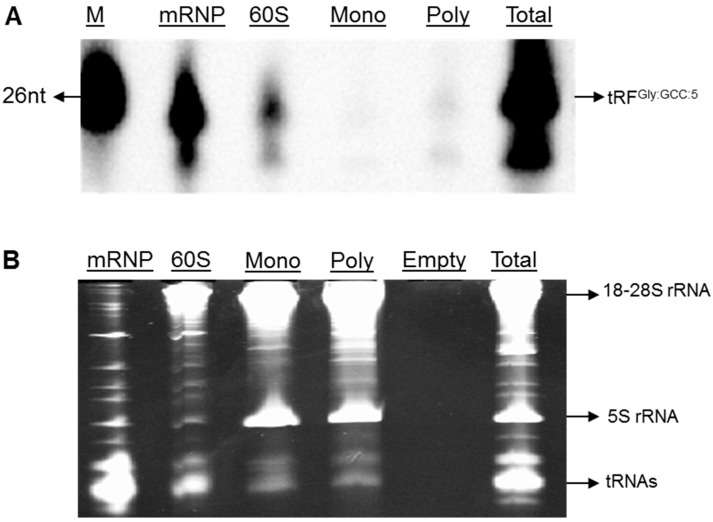
Non-polysomal association of biotinylated-tRF^Gly:GCC:5^ transfected into S2 cells. (**A**) tRF^Gly:GCC:5^ was biotinylated at nt-24 (Eurogentec, Belgium) and transfected into S2 cells. 48 h post-transfection with biotinylated-tRF^Gly:GCC:5^, S2 cytosolic cell lysates were layered over 5–70% sucrose density gradients. Following centrifugation at 27.000 rpm for 2 h 55 min at 4 °C in a Beckman SW28 rotor, fractions were collected from the top of the gradients using an ISCO density gradient system. Fractions were then pooled into 4 sub-groups based on their A_254_ readings; mRNP, 60S, monosome (mono) and polysome (poly). RNA extracted from the fractions or unfractionated cells (Total) were run on 12% denaturing polyacrylamide gel. Streptavidin-conjugated horse radish peroxidase was used to visualize the biotinylated tRFs; (**B**) Ethidium bromide staining of RNAs run on 12% denaturing polyacrylamide gel. M—Marker.

To solidify the non-polysomal localization of tRFs in S2 cells, we transfected S2 cells with biotinylated tRF^Gly:GCC:5^. Transfected tRFs were stable in S2 cells up to 72 h (Data not shown). The polysome profiling showed that the majority of biotinylated tRF^Gly:GCC:5^ fragments co-sediments with non-polysomal fractions similar to the in vivo products in embryos and S2 cells (Figure 3A). It is worth noting that a fraction of tRF associates with the polysomal fraction parallel to the deep-sequencing data.

### 3.3. tRNA-Derived Fragments are Shorter than Stress-Induced tRNA Cleavage Products

**Figure 4:**
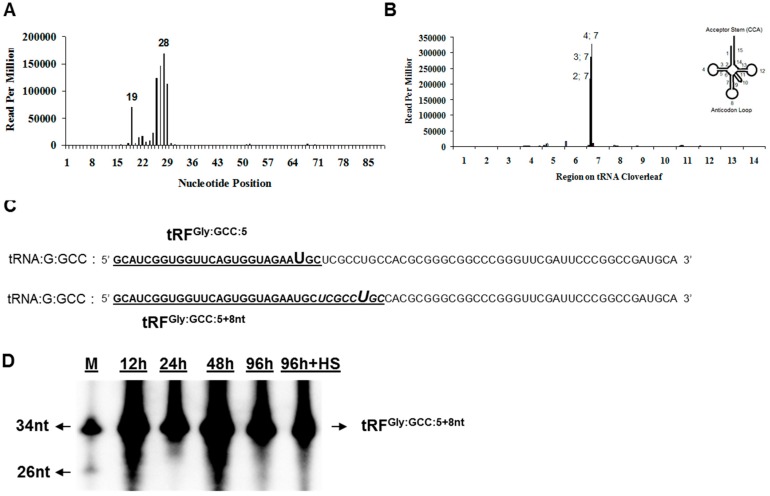
Three-foot-end-extended tRFs are not trimmed in S2 cells in the absence or presence of heat shock. (**A**) Three-foot-end cleavage site is relative to the 5’-end of tRNAs. All tRFs were aligned to the tRNA they originated from and 3’ cleavage site was determined relative to the 5’ end. The relative cloning frequency (read-per-million) was superimposed on a representative tRNA; (**B**) Each tRNA was divided into 15 main coordinates shown on a typical cloverleaf structure on the upper right corner. Each tRNA was folded using tRNAScan-SE [31] and the position of the 3’-end cleavage site was determined relative to each of the 15 coordinates. Normalized read-per-million cloning frequency of each fragment was plotted against the 15 positions. Nucleotide position is shown followed by the coordinate on the tRNA coverleaf (2;7 representing second nt on the 7th coordinate) (**C**) Nucleotide sequence of tRNA^Gly:GCC^. The sequence of tRF^Gly:GCC:5^ and tRF^Gly:GCC:5+8nt^ (which contains an 8-nt extension) are underlined. The 8-nt extended sequence is italicized. The biotinylated U residues are magnified. (**D**) Denaturing polyacrylamide analysis of tRF^Gly:GCC:5+8nt^ in S2 cells. S2 cells were transfected with biotinylated tRF^Gly:GCC:5+8nt^ and total RNAs isolated at the time indicated were resolved on 12% denaturing polyacrylamide gel. M—Marker; HS—heat shock.

sitRNA fragments are generated under stress conditions and their 3’ cleavage sites are located in the middle of the anticodon loop [30]. On the other hand, tRFs are relatively shorter in size (~26 nt) and their 3’ processing sites are poorly characterized. We extended our sitRNA-tRF comparison to their 3’-end cleavage site selection for further analysis. When all the reads are superimposed on a single representative tRNA, a great majority (95.74%) of tRFs are derived from the 5’-end sequences (Figure 1A). Despite a distinct 5’-end terminus (Figure 1A and Figure 2B), there appears to be heterogeneity in the 3’ cleavage site as we observed enrichment at nucleotide positions 26–28 (Figure 4A). We hypothesized that the secondary and/or tertiary structure, rather than the nucleotide sequence itself, might influence the 3’ cleavage site selection. To this end, we assigned 15 coordinates on individually folded tRNAs (Figure 4B) and aligned all tRNA-derived sequences relative to each coordinate. This analysis revealed that the 3’ processing site of tRF-5s is centred around the 5’ stem of the anticodon loop closer to the D arm (Figure 4B).

In addition to tRF-5s, we identified some tRFs that are complementary particularly to the mid-section or 3’-end sequences. Additionally, Northern blotting analysis showed the presence of sitRNA fragments longer than 26–28 nt in the mRNP fraction of both embryos and S2 cells (Figure 2D). Taken altogether, we hypothesized that tRF-5s may be cleaved as longer products (e.g., 35–40 nt sitRNA fragments), which may then be trimmed to an optimum length. To test this hypothesis, we transfected S2 cells with a control tRF^Gly:GCC:5^ and its 8-nt extended version (tRF^Gly:GCC:5+8nt^) (Figure 4C), assuming that the synthetic RNAs will be loaded on the same complexes as endogenous tRFs.

Interestingly, tRF^Gly:GCC:5+8nt^ was not shortened up to 96h post-transfection (Figure 4D). Trimming did not occur under heat shock conditions (Figure 4D, 96 h + HS) either, indicating that stress condition alone (e.g., heat shock) is not sufficient to induce trimming at the 3’end of tRF^Gly:GCC:5+8nt^ (Figure 4D, 96 h + HS).

### 3.4. tRFs Are Subject to Differential Expression During Development

**Figure 5:**
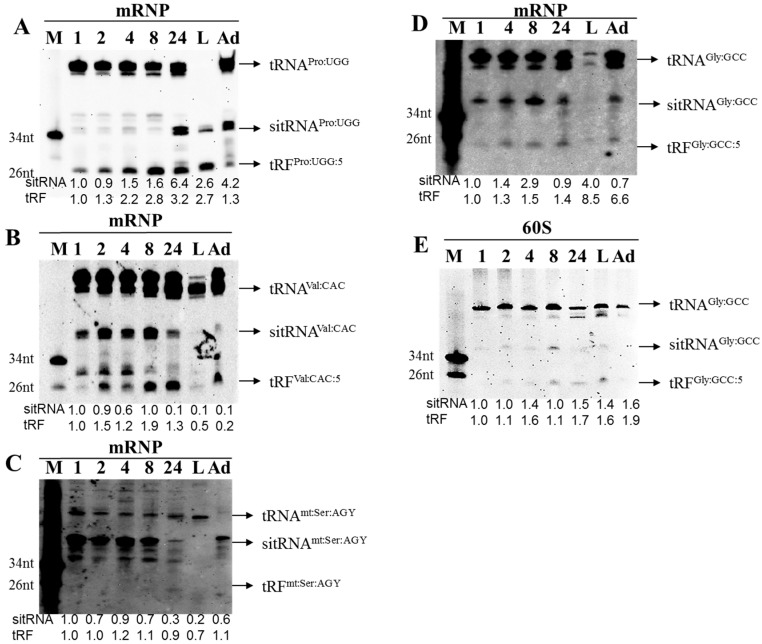
tRFs are differentially expressed during development in *Drosophila*. Polysome profiling of *Drosophila* samples of different developmental stages (1–24 h embryos; L—Larvae and Ad—Adult shown on top), RNA isolation and Northern blotting were performed as in Figure 2. RNA isolated from either mRNP fractions (**A**–**D**, 5 µg) or 60S fraction (**E**, 15 µg) was used in blotting. Data were normalized first against the mature tRNA levels and then to 1h embryos to calculate fold of induction for sitRNA and tRFs. The sitRNA and tRF expression ratios are based on signal intensity. Mature tRNA (tRNA^XXX^), stress-induced tRNA fragments (sitRNA^XXX^) and tRNA-derived fragments (tRF^XXX^) are shown to the right. M—Marker (size shown on the left).

Localization of tRFs within distinct intra-cytoplasmic locations (e.g., mRNP versus polysome) is quite interesting with respect to their biogenesis or functionality. We hypothesized that if tRFs are biologically functional regulatory molecules, they should be differentially expressed throughout the developmental stages. Thus, we subjected flies of various ages to polysome profiling to enrich for tRFs in mRNP and 60S fractions. The Northern blots showed that tRF^gly:GCC:5^ is up-regulated in 7–8 h embryos compared to 0–1 h embryos although the mature tRNA level remained nearly constant (Figure 5D, 1 vs. 8). The expression of tRF^Pro:UGG:5^ was quite prominent throughout the embryonic development and relatively less in adults (Figure 5A). Interestingly, sitRNAs (~35 nt) generated from this tRNA showed a differential biogenesis process in embryos up to 24 h. tRF^Val:CAC:5^ expression increased towards the later embryonic stages with a very low expression in larvae (Figure 5B). We detected a tRNA fragment of ~30 nt whose expression was reciprocal to that of tRF^Val:CAC:5^. The expression of tRF^mt:Ser:AGY:5^ was relatively lower in 24 h embryos (Figure 5C). More interestingly, the expression of sitRNAs gradually decreased towards the late embryonic stages. We also checked the expression of tRF^gly:GCC:5^ in RNAs extracted from the 60S fraction (Figure 5E). In accordance with the deep-sequencing data, this particular tRF was associated with the 60S fraction as well.

## 4. Discussion

In the present work, we demonstrate for the first time that tRF-5s are temporally and selectively expressed in 0–1 and 7–8 h *Drosophila* embryos under physiological conditions. More interestingly, tRF biogenesis and expression levels are likely to be modulated during early development in *Drosophila*. When we investigated their subcellular distribution, we observed that tRF-5s are associated primarily with non-polysomal fractions.

The tRFs detected in our studies are likely to be some regulatory small RNAs or simply the products of fluctuations in transcription or tRNA degradation. Based on the observation that the majority of the tRFs are detected from the 5’-end sequences, one could hypothesize that these fragments could be by-products of the 3’ → 5’ exonucleases that monitor tRNA quality in the nucleus [6]. Because we use the cytoplasmic extracts for polysome profiling, tRFs are unlikely to stem from the noise transcription products or the nuclear tRNA artifacts. The cytoplasmic extract could potentially contain tRNA degradation intermediates as part of the rapid tRNA decay [7]. However, this mechanism appears to be a general tRNA quality control pathway that monitors multiple hypomodified mature tRNAs. More importantly, the degradation takes place in the 5’ → 3’ orientation, which would generate, if any, 3’-end products rather than 5’-end ones [32]. We detect prominent 5’-end-derived tRNA fragments with no evidence for the smearing intermediates that are typically seen during RNA degradation. It is, however, intriguing that we were unable to detect tRFs from the 3’ arms except for a few tRNAs (Figure 1). A similar observation was reported by Olvedy et al. [33] in which 84.7% of tRFs were detected from the 5’-ends in a cohort of prostate cancer. Although the dominance of tRF-5s could carry some biological significance, we cannot eliminate sequencing bias. It is possible that chemical modifications enriched in the 3’ arms of tRNAs could conflict with the RT-based sequencing used in our methodology [34,35].

tRNA halves were initially reported in *Tetrahymena* and *Aspergillus* following amino acid starvation [25,36,37]. Later it was shown that tRNA cleavage is triggered in response to oxidative response and apoptosis in eukaryotes [4,8,32]. Therefore, tRNA fragmentation and its potential regulatory function were studied mostly under stress conditions or in proliferating and cancerous cells. Age-dependent modulation of tRF expression was previously reported [16]. Karaiskos et al. [16] provides very interesting data in that tRFs are associated with AGO1 and AGO2 in an age-dependent manner. Here we provide in vivo evidence that tRFs are differentially expressed in *Drosophila* embryos under physiological states. Differential and selective expression of embryonic and larval tRFs (Figure 5; Supporting Table S1) points to a potential biological role during the early development. Differential biogenesis (embryo versus larva, Figure 5) suggests that tRFs of varying sizes could be synthesized in response to different developmental cues. However, it requires further investigation to directly demonstrate the functional roles, if any, of tRFs of varying sizes in development.

There appear to be differences between tRFs reported here and sitRNA fragments. Firstly, stress-induced tRNA fragments are cleaved at or around the anti-codon loop [25,30]. However, we determined the cleavage site to be fairly upstream from the anticodon left arm at the juxtaposition with the D loop (Figure 4B). Secondly, the size of tRFs is quite shorter than that of the sitRNA fragments (Figure 2D). Additionally, tRF^Gly:GCC:5+8nt^ was not trimmed to its most abundant size of 26-28 nt both in the absence and presence of heat shock trea^tm^ent (Figure 4D), suggesting a biogenesis pathway requiring additional condition(s) or factor(s). Lastly, there is selectivity in the type and amount of tRFs under non-stress conditions whereas tRNA fragmentation is a general response under stress conditions. Our data suggest that selectivity exists not only among the tRNAs carrying different amino acids but also those carrying the same amino acid with a different anticodon (Supporting Table S1). However, sitRNA fragments co-sedimented with the mRNP fraction similar to the tRFs tested in this study (Figure 2F, HS). Thus, further studies should be conducted to reveal the mechanistic differences, if any, between sitRNA halves and tRFs.

Dicer-dependence [11], immunoprecipitation by Ago and Piwi proteins [13,16,18] and cytoplasmic localization [15] suggest that tRNA fragments may behave similar to miRNAs, siRNAs and piRNAs. However, tRF-1001 was shown to function through an unknown mechanism different from the miRNA pathway [12]. The data obtained from different species point to a conflict on polysomal association of tRFs. Reifur et al. [21] demonstrated that at least tRF^Glu-UCC^ is not associated with polysomes. However, in human cells, tRFs were shown to associate with polysomes and more importantly inhibit protein translation [20]. It is important to point out that both studies were conducted on a selected number of tRFs. Our genome-wide profiling data indicate that tRFs are primarily associated with non-polysomal fractions although we get some reads from polysomal fractions as well (Supporting Table S1). It would be interesting to check whether AGO-associated tRFs reported by Karaiskos et al. [16] localize to polysomal fractions. We interpret non-polysomal association of tRFs to mean that tRFs should mechanistically function differently from miRNAs. Although miRNAs are selectively associated with either of the four fractions in our experimental setting, tRFs are found at extremely low levels in polysomes (Table S1; Figure 2D,E). This lessens the possibility of a physical interaction between the overwhelming majority of tRFs and polysomes. tRFs particularly co-localize with complexes matching the size of 60S ribosomal subunit and mRNPs, suggesting that they are most likely localized away from the actively translating machinery. However, we cannot exclude the possibility that tRFs that co-purify with the 60S fraction (Figure 5E) could potentially form some complexes, which may associate with 40S, 43S, 48S or 60S ribosomal subunit complexes. Thus, it is possible that tRFs in *Drosophila* could regulate translation at the initiation step, similar to angiogenin-induced tRNA fragments [19] or tRFs in human cells [20].

## Supplementary Materials

The following are available online at www.mdpi.com/2073-4425/8/11/333/s1.
