# Supplementary material for: Differentially Expressed tRNA-Derived Small RNAs Co-Sediment Primarily with Non-Polysomal Fractions in Drosophila
Source: Genes (Basel). 2017 Nov 20;8(11):333. doi: 10.3390/genes8110333 (PMC5704246; doi:10.3390/genes8110333)
Supplement: Supplementary file 1 [file genes-08-00333-s001.pdf]

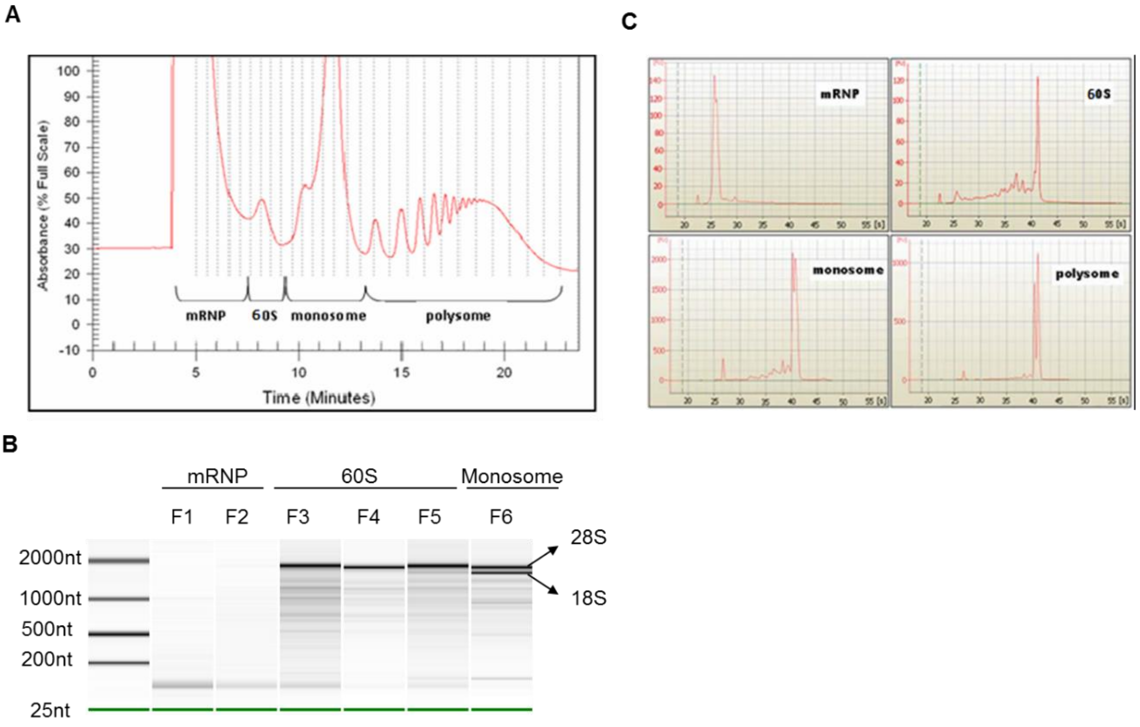

Supporting Figure S1. Fractionation of *Drosophila* embryos by polysome profiling and RNA QC. (**A**) Cytosolic (embryonic or S2) extracts were layered over 5-70% sucrose density gradients (See Experimental Procedures). Following ultracentrifugation, fractions were collected from the top of the gradients while reading absorbance at A_{254}. Based on the A_{254} reading, the fractions were pooled into 4 main fractions: mRNP, 60S, monosome and polysome. (**B**) rRNA content of subfractions. Following fractionation, total RNA was extracted from each fraction and run on bioanalyzer (Agilent, USA) to determine the rRNA content. An RNA ladder is shown on the left. (**C**) QC analysis of sub-fractions after pooling.

Supporting Table S1. Cloning frequency of tRFs matching individual tRNAs. All tRNA-derived fragments were aligned to 111 known *Drosophila* tRNA sequences. The cloning frequency of all tRNA-derived fragments matching to the same tRNA from unfractionated (Tot) or fractionated [mRNP, 60S, 80S and polysome] 0–1 and 7–8h embryos was summed and normalized as read-per-millon. tRNA:X-RA and CRXXXX-RA refer to the nomenclature used in flybase. Mt, mitochondrial

			**0-1h_Embryo**					**7-8h_Embryo**				
**tRNA_Name**	**CR_Code**	**Anticodon**	**mRNP**	**60S**	**80S**	**Polysome**	**Total**	**mRNP**	**60S**	**80S**	**Polysome**	**Total**
mt:tRNA:A-RA	CR34077-RA	tRNAAla	268	177	68	9	27	85	301	73	23	60
mt:tRNA:C-RA	CR34065-RA	tRNACys	148	95	32	47	28	810	162	60	63	91
mt:tRNA:D-RA	CR34071-RA	tRNAAsp	15	195	9	0	16	5	280	14	0	40
mt:tRNA:E-RA	CR34081-RA	tRNAGlu	2	55	2	0	3	5	102	4	0	7
mt:tRNA:F-RA	CR34082-RA	tRNAPhe	1830	290	186	160	60	1790	510	254	304	371
mt:tRNA:G-RA	CR34075-RA	tRNAGly	35	95	27	2	6	55	166	36	6	89
mt:tRNA:H-RA	CR34084-RA	tRNAHis	497	69	53	4	9	54	120	56	46	69
mt:tRNA:I-RA	CR34060-RA	tRNAIle	274	692	1034	76	210	362	739	909	98	245
mt:tRNA:K-RA	CR34070-RA	tRNALys	9019	2094	1267	342	124	37254	3742	2029	2858	2223
mt:tRNA:L:CUN-RA	CR34093-RA	tRNALeu	26	422	37	2	10	47	723	39	0	91
mt:tRNA:L:UUR-RA	CR34068-RA	tRNALeu	162	485	109	11	81	96	664	133	11	152
mt:tRNA:M-RA	CR34062-RA	tRNAMet	47	212	69	4	3	49	248	59	0	18
mt:tRNA:N-RA	CR34079-RA	tRNAAsn	367	313	58	4	8	281	647	102	11	42
mt:tRNA:P-RA	CR34088-RA	tRNAPro	189	90	70	11	1	118	130	67	11	49
mt:tRNA:Q-RA	CR34061-RA	tRNAGln	43	1221	44	2	6	87	934	58	6	71
mt:tRNA:R-RA	CR34078-RA	tRNAArg	275	220	217	22	36	136	431	428	46	164
mt:tRNA:S:AGY-RA	CR34080-RA	tRNASer	45496	665	5552	487	116	29872	1719	16790	2198	2198
mt:tRNA:S:UCN-RA	CR34091-RA	tRNASer	8	100	14	4	3	9	152	10	17	5
mt:tRNA:T-RA	CR34087-RA	tRNAThr	8	29	16	0	1	5	70	14	0	15
mt:tRNA:V-RA	CR34095-RA	tRNAVal	14	455	54	13	2	13	516	60	0	100
mt:tRNA:W-RA	CR34064-RA	tRNATrp(UCA)	13	90	23	0	4	10	141	17	0	20
mt:tRNA:Y-RA	CR34066-RA	tRNATyr	63	216	138	2	5	101	715	209	6	89
tRNA:CR30155-RA	CR30155-RA	tRNATrp(CCA)	232	38	60	9	25	12	19	17	0	21
tRNA:CR30199-RA	CR30199-RA	tRNAAla(CGC)	185	114	91	11	109	273	334	152	17	48
tRNA:CR30201-RA	CR30201-RA	tRNASer(TGA)	12	6	5	1	3	11	14	8	0	4
tRNA:CR30202-RA	CR30202-RA	tRNASer(TGA)	12	2	1	1	3	11	6	4	0	2
tRNA:CR30206-RA	CR30206-RA	tRNATrp(CCA)	263	162	148	24	29	13	47	44	0	45
tRNA:CR30232-RA	CR30232-RA	tRNAAla(TGC)	142	54	58	24	191	65	175	44	3	89
tRNA:CR30237-RA	CR30237-RA	tRNAGln(CTG)	438	141	44	51	37	94	112	64	29	68

tRNA:CR30239-RA	CR30239-RA	tRNAGlu(CTC)	365	1089	308	40	231	107	951	257	39	549
tRNA:CR30240-RA	CR30240-RA	tRNAGlu(TTC)	272	319	120	31	144	55	209	82	9	177
tRNA:CR30241-RA	CR30241-RA	tRNALeu(AAG)	22	24	8	0	4	7	11	3	0	5
tRNA:CR30254-RA	CR30254-RA	tRNAArg(AGA)	23	11	41	4	16	4	15	37	0	4
tRNA:CR30257-RA	CR30257-RA	tRNAThr(TGT)	57	19	21	7	9	39	24	18	0	14
tRNA:CR30298-RA	CR30298-RA	tRNAThr(AGT)	41	1	0	1	18	3	5	3	0	2
tRNA:CR30326-RA	CR30326-RA	tRNAVal(AAC)	41	7	2	8	35	1	7	1	0	56
tRNA:CR30407-RA	CR30407-RA	tRNAGly(TCC)	3393	623	27	249	230	751	238	45	884	273
tRNA:CR30449-RA	CR30449-RA	tRNAGlu(CTC)	64	15	15	5	45	10	12	8	7	69
tRNA:CR31070-RA	CR31070-RA	tRNAGln(TTG)	809	14818	657	105	67	499	10936	3974	849	989
tRNA:CR31130-RA	CR31130-RA	tRNALeu(TAA)	14	11	3	1	8	0	7	2	0	9
tRNA:CR31167-RA	CR31167-RA	tRNASer(GCT)	64	14	8	4	4	14	65	13	3	5
tRNA:CR31334-RA	CR31334-RA	tRNAThr(CGT)	56	24	24	11	51	77	46	13	6	41
tRNA:CR31356-RA	CR31356-RA	tRNAThr(TGT)	12	13	11	0	8	6	23	16	0	14
tRNA:CR31382-RA	CR31382-RA	tRNAAsp(GTC)	920	4742	1262	58	350	1181	5412	1558	146	1326
tRNA:CR31383-RA	CR31383-RA	tRNALeu(TAG)	7	6	1	0	7	1	4	2	0	3
tRNA:CR31416-RA	CR31416-RA	tRNAAsp(GTC)	558	4580	1185	47	312	1132	5270	1526	146	1247
tRNA:CR31494-RA	CR31494-RA	tRNAGly(TCC)	6540	1091	53	313	393	2353	441	74	2209	485
tRNA:CR31506-RA	CR31506-RA	tRNALeu(TAA)	6	1	3	1	0	0	2	1	0	0
tRNA:CR31570-RA	CR31570-RA	tRNAAla(AGC)	587	582	580	642	1919	520	1257	680	221	1208
tRNA:CR31577-RA	CR31577-RA	tRNAAla(AGC)	556	576	567	639	1916	516	1247	679	221	1198
tRNA:CR31631-RA	CR31631-RA	tRNAArg(CCT)	943	236	74	33	36	108	378	78	6	43
tRNA:CR31669-RA	CR31669-RA	tRNAGln(CTG)	583	5330	188	96	95	102	5721	748	23	430
tRNA:CR31940-RA	CR31940-RA	tRNAGln(CTG)	687	433	131	78	106	63	557	121	11	212
tRNA:CR31942-RA	CR31942-RA	tRNAArg(TCT)	0	0	0	0	0	0	0	0	0	0
tRNA:CR31946-RA	CR31946-RA	tRNAArg(TCT)	3	1	1	0	0	0	1	1	0	0
tRNA:CR31971-RA	CR31971-RA	tRNAAsp(GTC)	686	4566	1213	48	337	1139	5288	1529	143	1269
tRNA:CR32034-RA	CR32034-RA	tRNAThr(AGT)	28	0	0	1	9	1	2	1	0	1
tRNA:CR32129-RA	CR32129-RA	tRNAPro(AGG)	3828	4727	21122	473	884	1487	3603	27000	832	242
tRNA:CR32153-RA	CR32153-RA	tRNAMet(CAT)	61	18	19	6	10	17	11	3	11	43
tRNA:CR32173-RA	CR32173-RA	tRNAPro(CGG)	1927	1257	1221	67	536	4779	1024	2155	223	136
tRNA:CR32273-RA	CR32273-RA	tRNAMet(CAT)	36	13	14	6	9	9	7	4	11	41
tRNA:CR32287-RA	CR32287-RA	tRNACys(GCA)	181	5894	264	7	231	588	16740	522	17	574
tRNA:CR32303-RA	CR32303-RA	tRNACys(GCA)	45	100	56	2	23	31	217	71	0	16
tRNA:CR32312-RA	CR32312-RA	tRNACys(GCA)	2	0	0	0	2	0	0	1	0	1
tRNA:CR32359-RA	CR32359-RA	tRNALeu(CAG)	67	29	16	4	13	7	66	22	0	5

tRNA:CR32421-RA	CR32421-RA	tRNAVal(TAC)	142	114	37	7	18	27	39	33	0	20
tRNA:CR32449-RA	CR32449-RA	tRNACys(GCA)	11	10	4	2	4	3	9	7	0	2
tRNA:CR32460-RA	CR32460-RA	tRNAGlu(CTC)	255	424	294	20	189	119	372	285	42	406
tRNA:CR32481-RA	CR32481-RA	tRNAIle(TAT)	293	33	21	9	48	9	169	31	0	13
tRNA:CR32493-RA	CR32493-RA	tRNAGln(TTG)	780	2336	262	47	45	218	1993	1163	252	173
tRNA:CR32525-RA	CR32525-RA	tRNATyr(UAC)	97	61	23	18	71	102	421	80	17	162
tRNA:CR32748-RA	CR32748-RA	tRNAPhe(GAA)	64	100	47	9	32	22	86	17	6	175
tRNA:CR32826-RA	CR32826-RA	tRNAPro(CGG)	259	87	206	36	210	164	84	210	16	38
tRNA:CR33536-RA	CR33536-RA	tRNALeu(TAA)	6	2	2	1	2	0	3	1	0	3
tRNA:E4:62Ab-RA	CR32326-RA	tRNAGlu(CTC)	309	933	296	28	196	115	896	248	53	526
tRNA:G3:35Bd-RA	CR31981-RA	tRNAGly(GCC)	54606	29292	6701	2702	3925	73052	25655	12578	9425	18115
tRNA:G3:35Be-RA	CR31980-RA	tRNAGly(GCC)	54605	29287	6699	2702	3925	73052	25651	12578	9425	18115
tRNA:H:48F-RA	CR30252-RA	tRNAHis(GTG)	5305	1568	549	253	163	1657	2111	462	356	1135
tRNA:H:56E-RA	CR30233-RA	tRNAAla(TGC)	138	53	65	24	178	72	172	59	3	93
tRNA:I:49Fd-RA	CR30246-RA	tRNAIle(AAT)	10	17	10	7	19	10	39	11	0	42
tRNA:K2:42Ae-RA	CR32837-RA	tRNALys(CTT)	8225	3658	588	166	1008	3081	9991	1024	172	1540
tRNA:K5:29A-RA	CR31899-RA	tRNALys(TTT)	709	112	40	18	112	646	185	48	42	121
tRNA:K5:84ABb-RA	CR31489-RA	tRNALys(TTT)	9377	2488	1285	112	279	1231	5300	2706	624	742
tRNA:K5:87BC-RA	CR31197-RA	tRNALys(TTT)	672	129	73	32	211	716	207	101	33	160
tRNA:L:35C-RA	CR31831-RA	tRNALeu(TAA)	6	2	3	1	2	0	2	1	0	3
tRNA:L3:49Fa-RA	CR32841-RA	tRNALeu(CAA)	28	9	9	9	10	3	19	9	0	10
tRNA:L3:49Fb-RA	CR30508-RA	tRNALeu(CAA)	57	19	18	7	12	3	20	10	0	16
tRNA:M3:46A-RA	CR30003-RA	tRNAMet(CAT)	104	27	30	3	17	208	40	55	6	26
tRNA:M-i:61D-RA	CR32482-RA	tRNAMet(CAT)	226	32	30	6	18	226	46	57	6	58
tRNA:N5:60C-RA	CR33537-RA	tRNAAsn(GTT)	91	77	46	11	14	12	16	13	0	11
tRNA:P:3E-RA	CR32784-RA	tRNAPro(CGG)	270	266	2401	37	199	199	124	1783	25	30
tRNA:P:90Ca-RA	CR31567-RA	tRNAPro(TGG)	5551	1588	4197	60	552	11919	1609	7161	63	655
tRNA:Q:34E-RA	CR31838-RA	tRNAGln(CTG)	606	824	91	39	58	181	383	110	11	283
tRNA:R:12Ea-RA	CR32622-RA	tRNAArg(TCG)	199	82	59	17	19	14	26	29	2	19
tRNA:R:12Ee-RA	CR32624-RA	tRNAArg(TCG)	262	147	100	20	31	46	133	44	2	40
tRNA:R:12Ef-RA	CR32610-RA	tRNAArg(TCG)	246	140	95	19	28	46	127	41	2	40
tRNA:R:83AB-RA	CR31593-RA	tRNAArg(TCG)	60	22	12	6	8	4	11	7	0	5
tRNA:R2:84Fd-RA	CR31585-RA	tRNAArg(ACG)	158	54	83	4	26	41	33	35	0	33
tRNA:S2b:88A-RA	CR31476-RA	tRNASer(GCT)	101	16	27	2	5	35	77	49	9	9
tRNA:S4:12Ea-RA	CR32621-RA	tRNASer(CGA)	116	37	25	8	52	46	39	22	9	115
tRNA:S474:12Eh-RA	CR32615-RA	tRNASer(AGA)	115	37	25	8	51	43	37	22	9	115

tRNA:S7:64D-RA	CR32419-RA	tRNASer(AGA)	93	34	17	5	34	8	42	19	3	34
tRNA:S774:12Ec-RA	CR32612-RA	tRNASer(AGA)	75	29	14	3	22	7	36	13	3	31
tRNA:SeC-RA	CR30024-RA	tRNASeC(e)(TCA)	70	14	11	13	8	56	8	8	0	7
tRNA:T:90Cb-RA	CR31590-RA	tRNAThr(AGT)	109	22	12	10	37	6	15	8	0	23
tRNA:V3b:90BC-RA	CR31572-RA	tRNAVal(CAC)	63	142	78	16	13	471	313	280	86	74
tRNA:V3b:92Ba-RA	CR31214-RA	tRNAVal(CAC)	137	51	20	13	46	91	80	42	11	200
tRNA:V4:89BC-RA	CR31587-RA	tRNAVal(AAC)	5	2	2	1	5	0	3	1	0	5
tRNA:Y1:85Ac-RA	CR31434-RA	tRNATyr(GTA)	182	98	42	22	93	113	454	93	11	175
